# Purification of Gastric Mucosal ECL Cells from a Crude Elutriation Fraction

**DOI:** 10.1100/tsw.2002.852

**Published:** 2002-06-14

**Authors:** John Graham

**Affiliations:** School of Biomolecular Sciences, Liverpool John Moores University, UK

**Keywords:** gastric mucosa, ECL cells, OptiPrep^TM^, iodixanol, density barrier

## Abstract

Acid-secreting parietal cells from the gastric mucosa are widely studied as a model in studies on ion transport and the endocrine/paracrine ECL cells effectively control parietal cell function. Discontinuous gradients of iodixanol for the purification of ECL cells were subsequently simplified to the use of a density barrier. This technique is now commonly used following initial centrifugal elutriation.

**METHOD TYPE:** extraction, isolation, purification and separation

**SUB METHOD TYPE:** centrifugation

## INTRODUCTION

Investigations into the functioning and control of cells of the gastric mucosa are important in understanding the physiological activity of this tissue in both normal and clinical conditions. They have however a broader significance, since the gastric mucosa is widely recognized as an important model system for studying the functioning and control of any secretory epithelium.

Probably the most intensively studied cell is the acid-secreting parietal cell as this provides a very useful model for studying both the regulation of ion-transport and intracellular signaling pathways[1]. ECL cells are the main endocrine/paracrine cell type and they play an important role in controlling acid secretion[2].

This Protocol Article will describe the purification of ECL cells. The purification of parietal and chief cells is presented in a companion Protocol Article[3].

ECL cells have been purified by repeated centrifugal elutriation followed by banding in a discontinuous iodixanol gradient[1]. Subsequently the two layer discontinuous gradient was simplified to a density barrier[2] and it is this method that is presented in this Protocol Article.

## MATERIALS AND EQUIPMENT

OptiPrep™ (60% w/v, iodixanol)
OptiPrep™ diluent: 60 mg/ml BSA, 7.2 mM $MgCl_2$, 90 mM Hepes-NaOH pH 7.4
OptiPrep™ Working Solution (WS) of 50% (w/v) iodixanol: mix 5 vol of OptiPrep™ with 1 vol of Solution B
WS Diluent: 10 mg/ml BSA, 140 mM NaCl, 1.2 mM $MgSO_4$, 1 mM $CaCl_2$, 0.5 mM DTT, 1 mM glucose, 15 mM Hepes-NaOH, pH 7.4

Plastic conical centrifuge tubes (12–15 ml)
Syringe with metal cannula or plastic Pasteur pipette
Low-speed (temperature-controlled) centrifuge with swinging-bucket rotor

## METHOD

1. Disperse the gastric mucosal cells by Pronase digestion[4].
2. Partially purify the ECL cells by centrifugal elutriation[1].
3. Dilute WS with the WS Diluent to produce a solution of 10.8% (w/v) iodixanol (approx. equivalent to $\rho$ = 1.061 g/ml).
4. Layer 1 ml of the ECL cell containing suspension ($2 \times 10^6$ cells) over 10 ml of the iodixanol solution in a 15-ml centrifuge tube (see Note 1).
5. Centrifuge at 1,000 rpm for 5 min (at speed), using slow acceleration (400 rpm.min$^{-1}$) and no brake during deceleration.
6. The ECL cells band just above the 10.8% iodixanol layer (see Fig. 1).

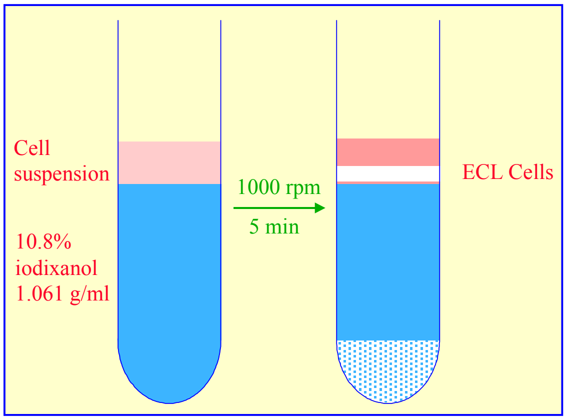

**FIGURE 1**. Isolation of gastric ECL cells on an iodixanol density barrier.

## NOTES

As this method isolates the least dense cell from a mixed population containing denser cells, it may be worthwhile considering an alternative flotation strategy. The cells should be suspended in a 15% (w/v) iodixanol solution (dilute WS with WS Diluent) and layered beneath the 10.8% iodixanol solution. This flotation strategy has been used very successfully for the isolation of other low-density cells from tissue digests (e.g., [5,6]).

## ACKNOWLEDGEMENTS

The author and TheScientificWorld wish to thank Axis-Shield PoC, AS, Oslo, Norway for their kind permission to adapt OptiPrep™ Application Sheet C31 in the preparation of this Protocol Article.
